# Association of Matrix Metalloproteinases Polymorphisms with Glaucoma Risk, Glaucoma Phenotype, and Response to Treatment with Selective Laser Trabeculoplasty or Latanoprost

**DOI:** 10.3390/ijms252413464

**Published:** 2024-12-16

**Authors:** Makedonka Atanasovska Velkovska, Katja Goričar, Tanja Blagus, Vita Dolžan, Barbara Cvenkel

**Affiliations:** 1 Department of Ophthalmology, University Medical Centre Ljubljana, Grablovičeva 46, 1000 Ljubljana, Slovenia; atmakedonka@yahoo.com; 2 Pharmacogenetics Laboratory, Institute of Biochemistry and Molecular Genetics, Faculty of Medicine, University of Ljubljana, Vrazov trg 2, 1000 Ljubljana, Slovenia; katja.goricar@mf.uni-lj.si (K.G.); tanja.blagus@mf.uni-lj.si (T.B.); vita.dolzan@mf.uni-lj.si (V.D.); 3 Faculty of Medicine, University of Ljubljana, Vrazov trg 2, 1000 Ljubljana, Slovenia

**Keywords:** glaucoma, latanoprost, matrix metalloproteinases, selective laser trabeculoplasty, single nucleotide polymorphisms

## Abstract

In open-angle glaucoma, the increase in intraocular pressure (IOP) is caused by an increased resistance to aqueous humour outflow in the trabecular meshwork. Since genetic variability of matrix metalloproteinase (MMP) genes may influence extracellular matrix remodelling, we investigated their association with glaucoma risk and/or response to treatment. The retrospective part of the study included patients with primary open-angle glaucoma and ocular hypertension (OHT); in the prospective part of the study, newly diagnosed patients with POAG or OHT were randomised to receive either latanoprost or selective laser trabeculoplasty (SLT) as the initial treatment. The reduction in IOP was measured 6 weeks after treatment. The following MMP single nucleotide polymorphisms were genotyped: *MMP2* rs243865, rs243849, and rs7201; *MMP3* rs3025058; *MMP9* rs17576, rs17577, rs20544, and rs2250889; *MMP14* rs1042704, rs1042704, and rs743257. Logistic regression was used to calculate odds ratios to assess the association between MMP polymorphism and risk of POAG or OHT, glaucoma phenotypes and response to treatment. Only carriers of the *MMP3* rs3025058 TT genotype had a significantly higher risk of OHT, more advanced glaucoma, and a higher C/D ratio in the additive and dominant models. None of the investigated MMP polymorphisms were associated with response to treatment with latanoprost and SLT in our study population.

## 1. Introduction

Glaucoma, a major cause of irreversible visual impairment and blindness, is characterised by the progressive loss of retinal ganglion cells and their axons. The pathogenesis of glaucoma is multifactorial, and several genetic and/or environmental factors interact to cause the disease [1,2]. Intraocular pressure (IOP) remains the most important risk factor for the disease, and its reduction is the only proven treatment that can prevent or delay the progression of glaucoma [3].

The increase in IOP in open-angle glaucoma is caused by an imbalance between the production of aqueous humour and its outflow, which is due to an increased resistance to the outflow of aqueous humour, mainly in the juxtacanalicular part of the trabecular meshwork [4]. The outflow resistance is generated by the extracellular matrix (ECM), which is continuously remodelled by various matrix metalloproteinases (MMPs) and tissue inhibitors of MMPs (TIMPs), which are constitutively expressed by the trabecular meshwork cells [5]. MMPs are a family of proteolytic enzymes with a wide range of functions, including cleavage of ECM components, proteinases, growth factors, cytokines, cell surface receptors, cell adhesion molecules, DNA repair enzymes, and mediators of apoptosis [6,7].

MMPs are important modulators of IOP homeostasis. Trabecular meshwork cells sense the stretching/distortion of the ECM caused by the increase in IOP and respond by upregulating their secretion of MMP-2, -3, and -14 while decreasing MMP-9 and TIMP-2 levels, thereby increasing the rate of ECM turnover and reducing resistance to aqueous outflow through the trabecular meshwork and restoring normal IOP [8,9,10,11,12]. Evidence from in vivo and in vitro studies using animal models and anterior segment explants and cell cultures indicates that the mechanism of IOP lowering by prostaglandin analogues also involves increased MMP expression in the trabecular meshwork and ciliary body, resulting in tissue remodelling that enhances uveoscleral and trabecular meshwork outflow [13,14].

IOP homeostasis is not the only aspect of glaucoma in which MMPs play a role. MMPs are expressed by various glial cells and neuronal cell types and may play a role in degenerative processes [15]. In animal models of glaucoma, MMP-9 in the retina had a negative impact on retinal ganglion cell survival, while the function of other MMPs is less known [15,16].

Genetic polymorphisms in the MMP genes and their regulatory regions may affect gene expression, enzyme stability or amino acid changes and may be associated with risk of glaucoma and/or response to treatment. However, most of the published literature only addressed the association of MMP single nucleotide polymorphisms (SNPs) with glaucoma risk and did not investigate the associations with glaucoma phenotype or response to treatment [17,18,19,20,21]. In addition, some MMP polymorphisms were not associated with glaucoma risk in one population, while the same SNP increased disease risk or even had a protective effect in another population [18,20,22,23].

The aim of our study was to investigate the impact of common MMP SNPs and their association with the risk of glaucoma, ocular hypertension, and association with glaucoma phenotype—e.g., disease severity, cup-to-disc (C/D) ratio, IOP, and central corneal thickness (CCT). In addition, we also investigated the association between selected SNPs and response to treatment with selective laser trabeculoplasty (SLT) and the prostaglandin analogue latanoprost.

## 2. Results

Patients’ characteristics

To assess the risk of glaucoma, we included 307 patients, of whom 235 had POAG and 72 had only OHT. Their clinical characteristics are presented in Table 1. Additionally, the control group consisted of 339 healthy blood donors with a median age of 49 (45–55) years, of whom 251 (74.0%) were male. There were more men in the control group than among the patients with glaucoma (*p* < 0.001), while the patients with POAG or OHT were older than the control group (*p* < 0.001).

To assess the association with treatment response, we included 52 patients treated with latanoprost and 51 patients treated with SLT. Their clinical characteristics are shown in Table 1. Patients treated with SLT were younger (*p* = 0.021), more often had OHT (*p* < 0.001), had lower C/D ratio in both the right and left eye (*p* = 0.014 and *p* = 0.003, respectively) and lower IOP in the left eye (*p* = 0.016).

Genotype frequencies

The genotype frequencies of selected polymorphisms in healthy controls, all patients, and patients treated with either latanoprost or SLT are presented in Supplementary Table S2. Among the MMP polymorphisms studied, the distribution of the *MMP14* rs1042703 genotypes in the control group was not in agreement with the Hardy–Weinberg equilibrium (HWE) (*p* = 0.037). It was therefore not included in the retrospective part of the analysis. In addition, *MMP2* rs243865 and *MMP9* rs2250889 were not in agreement with the HWE in patients treated with SLT and were therefore not included in the analysis of treatment response to latanoprost (Supplementary Table S1).

Retrospective study

### 2.1. Association Between Selected MMP Polymorphisms with the Risk of Glaucoma or Ocular Hypertension and the Glaucoma Phenotype

We did not find statistically significant associations between MMP polymorphisms and glaucoma (both POAG and OHT) (Supplementary Table S3). In the separate risk analysis for POAG (Table 2) and OHT (Table 3), we found a nominally significant association only between the *MMP3* polymorphism rs3025058 and the risk for OHT. Carriers of two polymorphic rs3025058 T alleles were more likely to have OHT (OR = 2.89; 95% CI = 1.30–6.41, *p* = 0.009), and the association remained nominally significant even after adjustment for age and sex (OR =2.96; 95% CI = 1.15–7.62, *p* = 0.025). In the dominant model, rs3025058 was nominally associated with higher OHT risk only in univariate analysis (OR = 2.38; 95% CI = 1.17–4.83, *p* = 0.017).

Additionally, significant differences in *MMP3* rs3025058 genotype distributions in the dominant model were observed between patients with different degrees of glaucomatous damage: carriers of two normal alleles were more likely to have advanced POAG, while carriers of two polymorphic alleles were more likely to have OHT (*p* = 0.002, Table 4).

The *MMP9* rs20544 CC genotype showed a nominal association with thicker CCT in the additive model (Padd = 0.041). Carriers of the *MMP3* rs3025505 genotype (-;-) had a greater C/D ratio in the additive (Padd = 0.026) and dominant models (Pdom = 0.012) (Table 5).

Prospective study

### 2.2. Association Between the Selected MMP Polymorphisms and the Response to Treatment

#### 2.2.1. Association Between the Selected MMP Polymorphisms and Response to Treatment with Latanoprost

After 6 weeks of treatment, 3 (5.8%) patients experienced a reduction in IOP of less than 15%, 17 (32.7%) patients experienced a reduction in IOP of between 15% and 30%, while 32 (61.5%) of patients experienced a reduction in IOP of more than 30%. The investigated MMP polymorphisms were not significantly associated with the response to latanoprost treatment (Table 6).

#### 2.2.2. Association Between the Selected MMP Polymorphisms and Response to Treatment with SLT

In the group treated with SLT, SLT was performed at a 360-degree angle with a median energy of each application of 0.9 (0.9–1.0) mJ for the right and the left eye, and the median number of spots was 90 (84–94) for the right eye and 90 (85–94) for the left eye. After 6 weeks of treatment, 7 (13.7%) patients experienced a reduction in IOP of less than 15%, 24 (47.1%) patients experienced a reduction in IOP of between 15% and 30%, while 20 (39.2%) of patients experienced a reduction in IOP of more than 30%. 

The investigated MMP polymorphisms were not significantly associated with the response to SLT treatment (Table 6).

## 3. Discussion

Elevation of IOP, which is the most common cause of glaucomatous damage, can be caused by increased outflow resistance of the trabecular meshwork. The outflow resistance is generated by the ECM, which is continuously remodelled by MMPs and their inhibitor TIPMs and is responsible for IOP homeostasis. In eyes with open-angle glaucoma, changes in the MMP–TIMP balance and reduced MMP activity in the aqueous humour of glaucoma patients have been found, both of which may promote abnormal ECM accumulation [24,25]. Samples of trabecular meshwork from patients with primary and secondary open-angle glaucoma showed an imbalance in the expression of MMPs and TIMPs, indicating a weaker reactivity to ECM accumulation in the trabecular meshwork compared to healthy control eyes [26]. This study investigated the associations of genetic polymorphisms in the regulatory regions of the *MMP* genes with the risk of glaucoma and response to treatment. The main findings of this study are that selected *MMP* SNPs may be associated with the risk of OHT but not POAG. However, associations with the degree of glaucomatous damage and glaucoma phenotype, such as CCT and the C/D ratio, were also observed.

### 3.1. Association of Selected SNPs with Risk of Glaucoma and OHT and Glaucoma Phenotype

In our study, selected *MMP* gene SNPs were associated with the risk of OHT but not with the risk of POAG. We found a significantly higher risk of OHT in carriers of the *MMP3* rs3025058 TT genotype. This SNP leads to a single adenine insertion (6A) or deletion (5A) at position -1171 in the 5′-UTR and influences the regulation of *MMP3* gene expression [27,28]. Functional studies have shown that the 6A allele is associated with twofold lower transcriptional activity, resulting in reduced gene expression [29]. This SNP was studied only in Greek patients with exfoliation with and without glaucoma and showed a similar distribution of genotypes and alleles between patients with exfoliation and controls [30]. Other studies reported the associations of the *MMP3* -1171 6A allele with the risk of Alzheimer’s disease [31] and its earlier onset [32].

Mossbock et al. [20] found no association between *MMP1* (rs1799750), *MMP2* (rs243865, also investigated in our study; rs243866), and *MMP9* (rs17576, also included in our study) polymorphisms and the presence of POAG or exfoliative glaucoma in the Caucasian population. In Polish patients with POAG, *MMP2* rs243865 TT and rs2285053 TT genotypes were associated with rim area in early glaucoma, suggesting a protective role in disease progression [18]. However, other studies [19,33] found a statistically significant increased risk of POAG associated with the *MMP1* rs1799757 2G/2G genotype and -1607 2G allele as well as for the *MMP9* rs3918249 (genotype C/T) and the -1562 T allele in Polish patients with POAG compared to healthy controls [19]. In POAG patients with these polymorphisms, the aqueous humour showed increased mRNA and protein expression of MMP1, MMP9 and MMP12, suggesting that the resulting altered MMP activity in the aqueous humour may promote the abnormal matrix remodelling characteristic of POAG [34]. Several studies investigated the association of *MMP* polymorphisms, particularly *MMP9*, with the risk of open-angle or angle-closure glaucoma in different populations [21,22,23,34]. Micheal et al. [28] reported that the *MMP1* rs1799750 (-1607 1G/2G) and *MMP9* rs17576 polymorphisms could be a potential sex-dependent risk factor for the development of POAG and primary angle-closure glaucoma in Pakistan, respectively. In the Russian population, the variant G allele of rs2250889 *MMP9* (which was also analysed in our study) was significantly associated with a higher risk of POAG [23] and exfoliative glaucoma [35], while the rs3918249 *MMP9* C allele decreased the risk of exfoliative glaucoma [35]. The *MMP9* rs3918249, rs3918242, and rs3918254 (not included in our study) polymorphisms were associated with a higher risk of developing POAG and primary angle-closure glaucoma in the North Indian [36] and Chinese populations [27], while rs3918249 may have a protective role for open-angle glaucoma in the Caucasian population [23,37]. Meta-analyses revealed an increased risk of glaucoma associated with the *MMP1* rs1799750 polymorphism [17,38] and a possible protective role of the *MMP9* rs17576 G>A polymorphism against the development of glaucoma in the Caucasian population [21].

In our study, we also investigated the association between selected SNPs and glaucoma phenotype, including IOP, C/D ratio, CCT, and disease severity, as defined by standard automated perimetry. We found that the *MMP3* rs3025058 TT genotype was associated with more advanced glaucoma and a higher C/D ratio in the additive and dominant models, while the *MMP9* rs520544 CC genotype was marginally associated with a thicker CCT. Markiewicz et al. [19] reported an association between *MMP1*-1607 1G/2G and *MMP9*-1562 C/T with reduced retinal nerve fibre thickness and reduced rim area with the *MMP12* -82 A/G polymorphism. Another study conducted with Polish patients with POAG found that the *MMP2* 735TT and -1306TT genotypes were significantly associated with a larger rim area (early glaucoma), suggesting a protective role against POAG [18]. In Russian patients, *MMP9* polymorphisms were associated with IOP in patients with POAG [23].

Nevertheless, further studies with larger sample sizes and including different ethnicities are needed to clarify the association between *MMP* polymorphisms and glaucoma risk and phenotype.

### 3.2. Association of Selected SNPs with Response to Treatment

Latanoprost [39,40,41,42] and SLT treatments [43,44] have been shown to affect the expression of MMPs that contribute to decreased resistance to aqueous humour outflow through the trabecular meshwork and uveoscleral pathway. We assessed the response to both treatments after 6 weeks, the time point at which both treatment modalities would show their maximum IOP-lowering effect.

#### 3.2.1. Response to Latanoprost

We found no statistically significant association between the selected MMP polymorphisms and the reduction in IOP after treatment with latanoprost. Ussa and co-workers [45] investigated whether the SNPs of the genes encoding MMP1, MMP2, MMP3, MMP9, and MMP17 were related to the response to latanoprost in 124 Spanish patients with open-angle glaucoma. Patients were categorised into three groups according to their response to 4 weeks of latanoprost treatment: Non-responders with a reduction in IOP of less than 15%, responders with a reduction in IOP of more than 15%, and hyper-responders with a reduction in IOP of more than 30% from baseline. They found that only the SNPs of the MMP1 gene (which were not investigated in our study) were significantly associated with non-response to latanoprost. In our study, we also defined poor response as a reduction in IOP of less than 15% and identified 5.8% non-responders versus 16.2% among Spanish patients, but we used IOP as a continuous variable in the association analysis to avoid data loss. In addition, response to latanoprost depends on the definition of non-response and patient adherence to treatment, which is generally better in patients participating in trials.

#### 3.2.2. Response to SLT

SLT is a widely used minimally invasive procedure to lower IOP in glaucoma patients. Response to SLT can vary from person to person [46], and efficacy is generally defined in studies as a reduction in IOP of at least 20% compared to pre-SLT levels [47,48,49]. In our study, we used the same criteria for non-response as for latanoprost, namely a reduction in IOP of less than 15%. Only 13.7% of our patients did not respond to SLT treatment. It is hypothesised that laser-induced stimulation of trabecular meshwork cells triggers changes in MMP activity that affect the remodelling process and that altered MMP activity could potentially influence the long-term efficacy of SLT. In trabecular meshwork cell culture, SLT increased the secretion of MMP3 [43]. Furthermore, in patients with exfoliative glaucoma, the reduction in IOP after SLT correlated with a decrease in the ratio between TIMP2 and MMP2 [44]. Polymorphisms in *MMP* genes have been linked to differences in MMP enzyme activity. In our study, we found no significant association between the MMP polymorphisms analysed and the reduction in IOP after treatment with SLT.

The limitations of our study are the small sample size and the analysis of only a few polymorphisms with known functions in *MMP* genes. There may be other polymorphisms involved in gene expression that alter the function of their enzymes. In addition, in terms of TIMPs, which are involved in the regulation of MMP activity [50], we did not investigate the potential influence of *TIMP* gene polymorphisms in our study. An even more complex analysis would be required to consider the combination of all polymorphisms on a specific gene expression as well as the influence of environmental factors.

Larger studies are needed that include all known genetic polymorphisms of the *MMP* and *TIMP* genes in patients treated with SLT and latanoprost, which could serve as potential genetic markers for glaucoma treatment decision-making.

## 4. Materials and Methods

The study was conducted in two parts. In the first retrospective part, we investigated the association between selected MMP polymorphisms with the risk of glaucoma or ocular hypertension and the glaucoma phenotype; in the second prospective part, we evaluated the association between the selected MMP polymorphisms and the response to treatment with SLT and latanoprost.

### 4.1. Study Participants

The study included 307 patients with primary open-angle glaucoma (POAG) or ocular hypertension (OHT) aged 40 years or more who were treated at the Department of Ophthalmology, University Medical Centre Ljubljana, Slovenia. They were all included in the retrospective study, while 103 patients were included in the prospective study of the response to treatment. Patients were diagnosed with ocular hypertension if the IOP exceeded 21 mmHg on several occasions, but the visual field was normal and reliable, and the optic disc was normal. Patients were diagnosed with POAG if the untreated IOP was above 21 mmHg in at least one eye and glaucomatous changes of the optic disc (rim thinning, loss of retinal nerve fibre layer) and/or repeatable visual field defects typical of glaucoma. The exclusion criteria included previous intraocular surgery (except in patients who had cataract surgery more than 6 months prior to inclusion), primary juvenile open-angle glaucoma or any other cause of optic neuropathy.

In terms of enrollment in the study, a medical history was taken from all patients, and a complete ophthalmological examination was performed (gonioscopy, anterior and posterior biomicroscopy, and CCT measurement). Intraocular pressure was measured using Goldmann applanation tonometry (1 pm ± 30 min). Glaucoma severity was defined as early (mean defect (MD) < 6 dB), moderate (MD from 6 to 12 dB) and advanced (MD > 12 dB) disease using standard automated perimetry (dynamic strategy, G-programme, Octopus 900 perimeter, Haag-Streit, Switzerland).

#### 4.1.1. Retrospective Part of the Study

Patients with POAG (n = 235) and OHT (n = 72) with at least 5 years of follow-up in the glaucoma unit of the Department of Ophthalmology were included in this part. Disease progression was determined from the medical records using a series of reliable visual fields using a commercially available trend-based analysis (EyeSuite™) and an assessment of optic disc and/or retinal nerve fibre layer changes.

A control group of 339 healthy, unrelated Slovenian blood donors without systemic diseases was included in the study. For the control group, only data on age and sex were available.

#### 4.1.2. Prospective Part of the Study

In the prospective part of the study, 103 newly detected treatment-naïve patients with POAG and OHT referred to the Glaucoma Unit of the Department of Ophthalmology were randomised to treatment with SLT or latanoprost once daily in the evening (Xalatan™, Upjohn EESV, Rivium Westlaan 142, 2909 LD Capelle aan den IJssel, The Netherlands).

SLT was performed on a 360°-angle circumference with non-overlapping applications, using energy to observe a fine (champagne-like) bubble formation. To prevent IOP spikes, a drop of 2% brimonidine tartrate (Bimanox, Jadran-Galenski laboratorij, 51000 Rijeka, Croatia) was administered before and after the laser procedure.

Treatment response to SLT and latanoprost was assessed after 6 weeks, measured with Goldmann applanation tonometry (1 pm ± 30 min). The reduction in IOP from baseline was expressed as an absolute change in IOP (mmHg) and as a relative change from baseline (in per cent). For comparison with previous studies, we also categorised patients into three groups: Non-responders with a reduction in IOP of less than 15%, responders between 15% and 30% and hyper-responders with a reduction in IOP of more than 30% from baseline.

### 4.2. DNA Isolation, SNP Selection and Genotyping

Peripheral blood samples were collected for DNA extraction. Genomic DNA was isolated using the E.Z.N.A.^®^ SQ II Blood DNA Kit (Omega Bio-tek, Inc., Norcross, GA, USA) according to the manufacturer’s instructions.

We searched for common, putatively functional polymorphisms in coding regions or 3’ and 5’ untranslated regions of MMP genes that could influence the composition of the ECM. In addition, some SNPs were selected based on previously published literature [18,20,28]. We selected only SNPs with a minor allele frequency greater than 0.05 in the European population. The predicted function of the polymorphisms was assessed using SNP Function Prediction, HaploReg v4.1 and GTEx [51]. The following SNPs were included: *MMP2* rs243865, rs243849 and rs7201; *MMP3* rs3025058; *MMP9* rs17576, rs17577, rs20544, and rs2250889; *MMP14* rs1042704, rs1042704, and rs743257. The predicted function of selected polymorphisms is presented in Supplementary Table S1.

Genotyping of all SNPs was performed using a competitive allele-specific assay according to the manufacturer’s instructions (LGC Genomics, UK). For all polymorphisms, 10% of the samples were genotyped in duplicates, and genotyping quality control criteria included a 100% duplicate call rate and a 95% SNP-wise call rate.

### 4.3. Statistical Analysis

Continuous and categorical variables were described with median and interquartile range (25–75%) and frequencies, respectively. As the variables were not normally distributed, non-parametric tests were used. Fisher’s exact test was used to compare the distribution of categorical variables, while non-parametric Mann–Whitney or Kruskal–Wallis tests were used to compare the distribution of continuous variables. Deviation from Hardy–Weinberg equilibrium (HWE) was evaluated using the standard chi-square test. Both dominant and additive genetic models were used in the analysis. The association of polymorphisms with glaucoma risk was assessed using logistic regression to calculate unadjusted and adjusted odds ratios (ORs) and 95% confidence intervals (CIs). For the IOP, CCT, and C/D ratio, data from the most affected eye were used for analysis. If a measurement was only available for one eye, the measurement from that eye was used for the analysis.

All statistical tests were two-sided. As eleven SNPs were included in the analyses, the Bonferroni correction was used to account for multiple comparisons and avoid false positives: *p*-values below 0.0045 were considered statistically significant, while *p*-values between 0.0045 and 0.050 were considered nominally significant. Statistical analyses were performed using IBM SPSS Statistics version 21.0 (IBM Corporation, Armonk, NY, USA).

## 5. Conclusions

In our study, selected *MMP* gene SNPs were not associated with the risk of POAG. However, in the separate analysis, which included only patients with OHT, we found a significantly higher risk of OHT in carriers of the *MMP3* rs3025058 TT genotype. In terms of the glaucoma phenotype, we found that the *MMP3* rs3025058 TT genotype was associated with more advanced glaucoma and a higher C/D ratio in the additive and dominant models, while the *MMP9* rs520544 CC genotype was marginally associated with a thicker CCT. We found no association between the investigated MMP polymorphisms and response to treatment with latanoprost and SLT.

## Figures and Tables

**Table 1:** Clinical characteristics of patients with POAG and OHT.

Characteristics	Category/Unit	POAG and OHT Patients N = 307	Latanoprost-Treated Patients N = 52	SLT-Treated Patients N = 51
Sex	Male, N (%)	139 (45.3)	28 (53.8)	24 (47.1)
	Female, N (%)	168 (54.7)	24 (46.2)	27 (52.9)
Age	Years, median (25–75%)	70 (64–78)	66 (56.8–74.8)	63 (55–68)
Diagnosis	OHT, N (%)	72 (23.5)	13 (25.0)	24 (47.1)
	Early POAG, N (%)	62 (20.2)	12 (23.1)	18 (35.3)
	Moderate POAG, N (%)	54 (17.6)	7 (13.5)	7 (13.7)
	Advanced POAG, N (%)	119 (38.8)	20 (38.5)	2 (3.9)
Family history of glaucoma	Yes, N (%)	98 (31.9)	11 (21.2)	17 (33.3)
IOP	Right eye, mmHg, median (25–75%)	19.71 (16.83–23)	24 (22–26)	23 (21–24)
	Left eye, mmHg, median (25–75%)	19.78 (16.7–23)	24 (22–27)	22 (21–24)
CCT	Right eye, µm, median (25–75%)	547 (521–574)	566.5 (533.5–590.3)	574 (543–603)
	Left eye, µm, median (25–75%)	548.5 (522.5–577)	574 (536–593)	571 (551–604)
C/D ratio	Right eye, median (25–75%)	0.8 (0.5–0.9)	0.6 (0.4–0.88)	0.5 (0.3–0.7)
	Left eye, median (25–75%)	0.8 (0.5–1)	0.7 (0.4–0.98)	0.5 (0.3–0.7)

CCT—central corneal thickness; C/D—cup-to-disc; IOP—intraocular pressure; OHT—ocular hypertension; POAG—primary open-angle glaucoma.

**Table 2:** Association of MMP polymorphisms with POAG risk.

Gene	SNP	Genotype	Controls (N = 339) N (%)	POAG Patients (N = 235) N (%)	OR (95% CI)	P	OR (95% CI) adj	Padj
*MMP14*	rs1042704	GG	217 (64)	151 (64.3)	Reference		Reference	
		GA	108 (31.9)	72 (30.6)	0.96 (0.67–1.38)	0.817	0.74 (0.36–1.51)	0.408
		AA	14 (4.1)	12 (5.1)	1.23 (0.55–2.74)	0.609	0.84 (0.17–4.19)	0.829
		GA + AA	122 (36)	84 (35.7)	0.99 (0.70–1.40)	0.952	0.75 (0.38–1.49)	0.412
	rs743257	CC	86 (25.4)	51 (21.7)	Reference		Reference	
		CT	161 (47.5)	128 (54.5)	1.34 (0.88–2.03)	0.168	1.12 (0.49–2.58)	0.786
		TT	92 (27.1)	56 (23.8)	1.03 (0.64–1.66)	0.915	1.24 (0.49–3.15)	0.649
		CT + TT	253 (74.6)	184 (78.3)	1.23 (0.83–1.82)	0.311	1.16 (0.53–2.55)	0.707
*MMP2*	rs243865	CC	201 (59.3)	142 (60.4)	Reference		Reference	
		CT	125 (36.9)	82 (34.9)	0.93 (0.65–1.32)	0.68	0.91 (0.46–1.81)	0.794
		TT	13 (3.8)	11 (4.7)	1.20 (0.52–2.75)	0.671	0.71 (0.16–3.24)	0.660
		CT + TT	138 (40.1)	93 (39.6)	0.95 (0.68–1.34)	0.785	0.89 (0.46–1.71)	0.720
	rs243849	CC	250 (73.7)	170 (72.3)	Reference		Reference	
		CT	79 (23.3)	57 (24.3)	1.06 (0.72–1.57)	0.767	1.09 (0.52–2.32)	0.815
		TT	10 (2.9)	8 (3.4)	1.18 (0.46–3.04)	0.737	1.96 (0.23–16.78)	0.540
		CT + TT	89 (26.3)	65 (27.7)	1.07 (0.74–1.56)	0.709	1.15 (0.55–2.38)	0.710
	rs7201	AA	127 (37.5)	84 (35.7)	Reference		Reference	
		AC	153 (45.1)	108 (46)	1.07 (0.74–1.54)	0.73	0.95 (0.45–1.99)	0.882
		CC	59 (17.4)	43 (18.3)	1.1 (0.68–1.78)	0.692	2.45 (0.98–6.16)	0.056
		AC + CC	212 (62.5)	151 (64.3)	1.08 (0.76–1.52)	0.675	1.27 (0.64–2.50)	0.499
*MMP9*	rs17576	AA	136 (40.1)	85 (36.2)	Reference		Reference	
		AG	164 (48.4)	120 (51.1)	1.17 (0.82–1.68)	0.389	1.04 (0.52–2.09)	0.901
		GG	39 (11.5)	30 (12.8)	1.23 (0.71–2.13)	0.457	0.62 (0.21–1.81)	0.381
		AG + GG	203 (59.9)	150 (63.8)	1.18 (0.84–1.67)	0.339	0.94 (0.48–1.82)	0.849
	rs2250889	CC	311 (91.7)	212 (90.2)	Reference		Reference	
		CG + GG	28 (8.3)	23 (9.8)	1.21 (0.68–2.15)	0.527	1.28 (0.40–4.06)	0.679
	rs17577	GG	238 (70.2)	161 (68.5)	Reference		Reference	
		GA	97 (28.6)	67 (28.5)	1.02 (0.71–1.48)	0.912	0.97 (0.47–1.99)	0.930
		AA	4 (1.2)	7 (3)	2.59 (0.75–8.98)	0.134	3 (0.34–26.68)	0.326
		GA + AA	101 (29.8)	74 (31.5)	1.08 (0.76–1.55)	0.664	1.05 (0.52–2.11)	0.888
	rs20544	CC	70 (20.6)	45 (19.1)	Reference		Reference	
		CT	160 (47.2)	123 (52.3)	1.20 (0.77–1.86)	0.428	1.32 (0.55–3.17)	0.538
		TT	109 (32.2)	67 (28.5)	0.96 (0.59–1.55)	0.856	1.05 (0.41–2.71)	0.922
		CT + TT	269 (79.4)	190 (80.9)	1.10 (0.72–1.67)	0.659	1.21 (0.53–2.77)	0.658
*MMP3*	rs3025058	--	94 (27.7)	60 (25.5)	Reference		Reference	
		-T	167 (49.3)	117 (49.8)	1.10 (0.74–1.64)	0.649	1.41 (0.63–3.16)	0.405
		TT	78 (23.0)	58 (24.7)	1.17 (0.73–1.86)	0.524	1.29 (0.51–3.29)	0.590
		-T + TT	245 (72.3)	175 (74.5)	1.12 (0.77–1.63)	0.559	1.37 (0.64–2.95)	0.420

Adj—Adjustment for age and sex.

**Table 3:** Association of MMP polymorphisms with the risk for OHT without POAG.

Gene	SNP	Genotype	Controls (N = 339) N (%)	OHT Without POAG (N = 72) N (%)	OR (95% CI)	P	OR (95% CI) adj	Padj
*MMP14*	rs1042704	GG	217 (64)	46 (63.9)	Reference		Reference	
		GA	108 (31.9)	21 (29.2)	0.92 (0.52–1.61)	0.765	0.80 (0.40–1.60)	0.526
		AA	14 (4.1)	5 (6.9)	1.68 (0.58–4.91)	0.339	2.66 (0.73–9.65)	0.137
		GA + AA	122 (36)	26 (36.1)	1.01 (0.59–1.71)	0.984	0.96 (0.50–1.84)	0.907
	rs743257	CC	86 (25.4)	16 (22.2)	Reference		Reference	
		CT	161 (47.5)	30 (41.7)	1.00 (0.52–1.94)	0.996	0.72 (0.33–1.60)	0.421
		TT	92 (27.1)	26 (36.1)	1.52 (0.76–3.02)	0.234	1.11 (0.48–2.58)	0.801
		CT + TT	253 (74.6)	56 (77.8)	1.19 (0.65–2.18)	0.575	0.86 (0.42–1.78)	0.686
*MMP2*	rs243865	CC	201 (59.3)	42 (58.3)	Reference		Reference	
		CT	125 (36.9)	29 (40.3)	1.11 (0.66–1.87)	0.695	0.86 (0.45–1.66)	0.659
		TT	13 (3.8)	1 (1.4)	0.37 (0.05–2.89)	0.342	0.23 (0.02–2.50)	0.225
		CT + TT	138 (40.1)	30 (41.7)	1.04 (0.62–1.74)	0.881	0.79 (0.42–1.51)	0.478
	rs243849	CC	250 (73.7)	48 (66.7)	Reference		Reference	
		CT	79 (23.3)	23 (31.9)	1.52 (0.87–2.65)	0.143	1.25 (0.63–2.52)	0.523
		TT	10 (2.9)	1 (1.4)	0.52 (0.07–4.16)	0.539	1.23 (0.13–11.71)	0.854
		CT + TT	89 (26.3)	24 (33.3)	1.40 (0.81–2.43)	0.223	1.25 (0.64–2.47)	0.515
	rs7201	AA	127 (37.5)	25 (34.7)	Reference		Reference	
		AC	153 (45.1)	35 (48.6)	1.16 (0.66–2.04)	0.602	1.15 (0.58–2.31)	0.688
		CC	59 (17.4)	12 (16.7)	1.03 (0.49–2.20)	0.932	1.06 (0.42–2.65)	0.906
		AC + CC	212 (62.5)	47 (65.3)	1.13 (0.66–1.92)	0.662	1.13 (0.59–2.16)	0.721
*MMP9*	rs17576	AA	136 (40.1)	36 (50)	Reference		Reference	
		AG	164 (48.4)	29 (40.3)	0.67 (0.39–1.15)	0.143	0.58 (0.30–1.14)	0.116
		GG	39 (11.5)	7 (9.7)	0.68 (0.28–1.64)	0.389	0.61 (0.22–1.67)	0.332
		AG + GG	203 (59.9)	36 (50)	0.67 (0.40–1.12)	0.124	0.59 (0.31–1.11)	0.100
	rs2250889	CC	311 (91.7)	66 (91.7)	Reference		Reference	
		CG + GG	28 (8.3)	6 (8.3)	1.01 (0.40–2.54)	0.984	0.71 (0.20–2.52)	0.593
	rs17577	GG	238 (70.2)	56 (77.8)	Reference		Reference	
		GA	97 (28.6)	14 (19.4)	0.61 (0.33–1.15)	0.129	0.60 (0.28–1.27)	0.182
		AA	4 (1.2)	2 (2.8)	2.13 (0.38–11.89)	0.391	1.58 (0.17–14.83)	0.691
		GA + AA	101 (29.8)	16 (22.2)	0.67 (0.37–1.23)	0.198	0.64 (0.31–1.33)	0.232
	rs20544	CC	70 (20.6)	11 (15.3)	Reference		Reference	
		CT	160 (47.2)	30 (41.7)	1.19 (0.57–2.52)	0.643	1.04 (0.42–2.53)	0.940
		TT	109 (32.2)	31 (43.1)	1.81 (0.85–3.83)	0.121	1.84 (0.74–4.53)	0.187
		CT + TT	269 (79.4)	61 (84.7)	1.44 (0.72–2.89)	0.300	1.34 (0.59–3.07)	0.484
*MMP3*	rs3025058	--	94 (27.7)	10 (13.9)	Reference		Reference	
		-T	167 (49.3)	38 (52.8)	2.14 (1.02–4.49)	0.044	1.77 (0.73–4.25)	0.205
		TT	78 (23.0)	24 (33.3)	2.89 (1.30–6.41)	0.009	2.96 (1.15–7.62)	0.025
		-T + TT	245 (72.3)	62 (86.1)	2.38 (1.17–4.83)	0.017	2.13 (0.92–4.89)	0.076

Adj—Adjustment for age and sex.

**Table 4:** Association of MMP polymorphisms with the degree of glaucomatous damage.

Gene	SNP	Genotype	OHT (N = 72)	Early POAG (N = 62)	Moderate POAG (N = 54)	Advanced POAG (N = 119)	P
*MMP14*	rs1042704	GG	46 (23.4)	41 (20.8)	31 (15.7)	79 (40.1)	Padd = 0.196
		GA	21 (22.6)	18 (19.4)	23 (24.7)	31 (33.3)	
		AA	5 (29.4)	3 (17.6)	0 (0)	9 (52.9)	
		GA + AA	26 (23.6)	21 (19.1)	23 (20.9)	40 (36.4)	Pdom = 0.702
	rs743257	CC	16 (23.9)	14 (20.9)	9 (13.4)	28 (41.8)	Padd = 0.363
		CT	30 (19)	32 (20.3)	30 (19)	66 (41.8)	
		TT	26 (31.7)	16 (19.5)	15 (18.3)	25 (30.5)	
		CT + TT	56 (23.3)	48 (20)	45 (18.8)	91 (37.9)	Pdom = 0.792
*MMP2*	rs243865	CC	42 (22.8)	33 (17.9)	37 (20.1)	72 (39.1)	Padd = 0.462
		CT	29 (26.1)	26 (23.4)	16 (14.4)	40 (36)	
		TT	1 (8.3)	3 (25)	1 (8.3)	7 (58.3)	
		CT + TT	30 (24.4)	29 (23.6)	17 (13.8)	47 (38.2)	Pdom = 0.408
	rs243849	CC	48 (22)	41 (18.8)	40 (18.3)	89 (40.8)	Padd = 0.625
		CT	23 (28.8)	18 (22.5)	12 (15)	27 (33.8)	
		TT	1 (11.1)	3 (33.3)	2 (22.2)	3 (33.3)	
		CT + TT	24 (27)	21 (23.6)	14 (15.7)	30 (33.7)	Pdom = 0.485
	rs7201	AA	25 (22.9)	19 (17.4)	22 (20.2)	43 (39.4)	Padd = 0.950
		AC	35 (24.5)	31 (21.7)	22 (15.4)	55 (38.5)	
		CC	12 (21.8)	12 (21.8)	10 (18.2)	21 (38.2)	
		AC + CC	47 (23.7)	43 (21.7)	32 (16.2)	76 (38.4)	Pdom = 0.728
*MMP9*	rs17576	AA	36 (29.8)	21 (17.4)	21 (17.4)	43 (35.5)	Padd = 0.553
		AG	29 (19.5)	34 (22.8)	26 (17.4)	60 (40.3)	
		GG	7 (18.9)	7 (18.9)	7 (18.9)	16 (43.2)	
		AG + GG	36 (19.4)	41 (22)	33 (17.7)	76 (40.9)	Pdom = 0.202
	rs2250889	CC	66 (23.7)	56 (20.1)	49 (17.6)	107 (38.5)	Pdom = 0.992
		CG + GG	6 (20.7)	6 (20.7)	5 (17.2)	12 (41.4)	
	rs17577	GG	56 (25.8)	42 (19.4)	34 (15.7)	85 (39.2)	Padd = 0.296
		GA	14 (17.3)	16 (19.8)	19 (23.5)	32 (39.5)	
		AA	2 (22.2)	4 (44.4)	1 (11.1)	2 (22.2)	
		GA + AA	16 (17.8)	20 (22.2)	20 (22.2)	34 (37.8)	Pdom = 0.305
	rs20544	CC	11 (19.6)	11 (19.6)	9 (16.1)	25 (44.6)	Padd = 0.444
		CT	30 (19.6)	32 (20.9)	30 (19.6)	61 (39.9)	
		TT	31 (31.6)	19 (19.4)	15 (15.3)	33 (33.7)	
		CT + TT	61 (24.3)	51 (20.3)	45 (17.9)	94 (37.5)	Pdom = 0.789
*MMP3*	rs3025058	--	10 (14.3)	7 (10.0)	15 (21.4)	38 (54.3)	Padd = 0.014
		-T	38 (24.5)	37 (23.9)	24 (15.5)	56 (36.1)	
		TT	24 (29.3)	18 (22.0)	15 (18.3)	25 (30.5)	
		-T + TT	62 (26.2)	55 (23.2)	39 (16.5)	81 (34.2)	Pdom = 0.002

Add—additive model; dom—dominant model.

**Table 5:** Association of selected polymorphisms with IOP, CCT, and C/D ratio.

Gene	SNP	Genotype	IOP Max Median (25–75%)	P	CCT Min Median (25–75%)	P	C/D Max Median (25–75%)	P
*MMP14*	rs1042704	GG	20.64 (17.57–23.42)	Padd = 0.562	545 (521–573)	Padd = 0.636	0.9 (0.6–1)	Padd = 0.863
		GA	20.54 (17.26–24)		538 (518–577.5)		0.8 (0.6–1)	
		AA	22 (16.96–25.1)		530 (507.5–568)		1 (0.35–1)	
		GA + AA	20.93 (17.28–24)	Pdom = 0.839	537.5 (517.25–576)	Pdom = 0.578	0.8 (0.5–1)	Pdom = 0.595
	rs743257	CC	21.19 (17.46–24)	Padd = 0.611	541 (510–573)	Padd = 0.563	0.9 (0.6–1)	Padd = 0.114
		CT	20.33 (17.35–23.45)		546.5 (520–574)		0.9 (0.6–1)	
		TT	20.5 (17.6–23.48)		539 (520–570)		0.8 (0.5–1)	
		CT + TT	20.43 (17.51–23.38)	Pdom = 0.321	543 (520–573)	Pdom = 0.438	0.9 (0.6–1)	Pdom = 0.818
*MMP2*	rs243865	CC	20.8 (17.22–23.46)	Padd = 0.716	540 (518–573)	Padd = 0.065	0.9 (0.6–1)	Padd = 0.195
		CT	21 (17.72–24)		553 (530–576)		0.8 (0.6–1)	
		TT	19.64 (17.64–23.64)		533.5 (512.75–556)		1 (0.8–1)	
		CT + TT	20.83 (17.72–24)	Pdom = 0.438	551 (524–574)	Pdom = 0.133	0.8 (0.6–1)	Pdom = 0.632
	rs243849	CC	20.93 (17.58–24)	Padd = 0.930	542 (520–571.25)	Padd = 0.840	0.9 (0.6–1)	Padd = 0.822
		CT	20.15 (17.21–24)		545 (518–576.75)		0.8 (0.5–1)	
		TT	21 (18.15–23.17)		544 (515.5–591)		0.8 (0.55–1)	
		CT + TT	20.23 (17.26–23.86)	Pdom = 0.792	545 (518–580)	Pdom = 0.919	0.8 (0.5–1)	Pdom = 0.533
	rs7201	AA	20.82 (17.48–23.91)	Padd = 0.799	540 (514.5–573)	Padd = 0.609	0.9 (0.6–1)	Padd = 0.890
		AC	20.43 (17.3–23.15)		548 (521–574)		0.9 (0.6–1)	
		CC	21 (17.67–24.19)		540 (520–571)		0.8 (0.6–1)	
		AC + CC	20.85 (17.54–24)	Pdom = 0.749	545 (521–573.25)	Pdom = 0.343	0.9 (0.6–1)	Pdom = 0.762
*MMP9*	rs17576	AA	21 (17.62–24)	Padd = 0.861	544 (521–574)	Padd = 0.091	0.8 (0.5–1)	Padd = 0.314
		AG	20.43 (17.34–24)		538 (513–568.5)		0.9 (0.6–1)	
		GG	20.82 (17.41–23.77)		557 (528.5–582)		0.9 (0.7–1)	
		AG + GG	20.61 (17.35–23.87)	Pdom = 0.599	542.5 (517.75–573)	Pdom = 0.423	0.9 (0.6–1)	Pdom = 0.159
	rs2250889	CC	20.83 (17.49–24)	Pdom = 0.477	543 (520–573)	Pdom = 0.784	0.9 (0.6–1)	Pdom = 0.303
		CG + GG	21 (17.41–22.85)		541 (513–578)		0.9 (0.65–1)	
	rs17577	GG	21 (17.59–24)	Padd = 0.683	544 (520–572.5)	Padd = 0.124	0.8 (0.55–1)	Padd = 0.174
		GA	20.13 (17.44–23)		538 (515–575)		0.9 (0.6–1)	
		AA	21 (16.74–25.91)		565 (552–587.5)		0.8 (0.5–0.95)	
		GA + AA	20.2 (17.34–23)	Pdom = 0.463	541.5 (518–577.25)	Pdom = 0.953	0.9 (0.6–1)	Pdom = 0.117
	rs20544	CC	20.61 (17.6–23.14)	Padd = 0.725	554 (527.5–579.5)	Padd = 0.041	0.9 (0.6–1)	Padd = 0.189
		CT	21 (17.23–24)		538 (511.5–569)		0.9 (0.6–1)	
		TT	20.93 (17.66–24)		551.5 (521.75–574)		0.8 (0.5–1)	
		CT + TT	21 (17.46–24)	Pdom = 0.728	541 (519–571)	Pdom = 0.096	0.8 (0.6–1)	Pdom = 0.304
*MMP3*	rs3025058	--	19.35 (17.12–23.35)	Padd = 0.056	538.5 (519.75–566.25)	Padd = 0.406	0.9 (0.78–1.0)	Padd = 0.026
		-T	20.54 (17.46–23.5)		542 (519–574)		0.8 (0.6–1.0)	
		TT	21.42 (18.25–24.02)		547.5 (520.75–580)		0.7 (0.5–1.0)	
		-T + TT	21 (17.59–24)	Pdom = 0.215	544 (520–576)	Pdom = 0.261	0.8 (0.6–1.0)	Pdom = 0.012

Add—additive model; dom—dominant model.

**Table 6:** Association of MMP polymorphisms with IOP reduction after latanoprost and SLT treatment.

Gene	SNP	Genotypes	IOP Change (mmHg) Median (25–75%)	P	Relative IOP Change (%) Median (25–75%)	P	IOP Change (mmHg) Median (25–75%)	P	Relative IOP Change (%) Median (25–75%)	P
			Latanoprost Treatment				SLT Treatment			
*MMP14*	rs1042703	TT	−8 (−10.8 to −6)	0.611	−31.4 (−41.2 to −25.3)	0.827	−7 (−8 to −4)	0.334	−26.9 (−33.3 to −19)	0.319
		TC + CC	−8 (−11.5 to −6)		−35.4 (−36.4 to −20.8)		−5.5 (−7.8 to −4)		−23.4 (−32.6 to −14.9)	
	rs1042704	GG	−8 (−11.3 to −6)	Padd = 0.339	−32.8 (−41.7 to −24.7)	Padd = 0.470	−6 (−8 to −4)	Padd = 0.408	−25 (−31.8 to −19)	Padd = 0.710
		GA	−8 (−10 to −6)		−33.3 (−39.9 to −27.3)		−6 (−7.5 to −4)		−26.1 (−35.5 to −16.7)	
		AA	−6 (−9 to −4)		−22.2 (−36.3 to −17.8)		−7 (−9 to −7)		−29.2 (−33.3 to −29.2)	
		GA + AA	−7.5 (−10 to −6)	Pdom = 0.444	−31.8 (−38.8 to −22.9)	Pdom = 0.735	−7 (−7.8 to −4)	Pdom = 0.666	−28 (−34.6 to −17)	Pdom = 0.626
	rs743257	CC	−8 (−10.5 to −6)	Padd = 0.814	−34.1 (−39 to −21)	Padd = 0.786	−6.5 (−7.8 to −4.3)	Padd = 0.692	−27.7 (−34.4 to −17.5)	Padd = 0.572
		CT	−8 (−10.8 to −6)		−30.8 (−40.1 to −23.6)		−6 (−7 to −4)		−26.1 (−30.4 to −18.2)	
		TT	−8.5 (−10.8 to −7)		−33.9 (−41.4 to −28.8)		−7 (−8.8 to −4)		−28.7 (−35.3 to −17.8)	
		CT + TT	−8 (−10.8 to −6)	Pdom = 0.653	−31.4 (−41.2 to −25.3)	Pdom = 0.890	−6 (−8 to −4)	Pdom = 0.797	−26.9 (−31.8 to −18.2)	Pdom = 0.576
*MMP2*	rs243865	CC	−8 (−11 to −6)	Padd = 0.303	−31.8 (−41.3 to −27.6)	Padd = 0.243	NA	NA	NA	NA
		CT	−6.5 (−9.5 to −6)		−27.9 (−35.7 to −20.8)		NA	NA	NA	NA
		TT	−10 (−12 to −10)		−36.4 (−41.4 to −36.4)		NA	NA	NA	NA
		CT + TT	−8 (−10 to −6)	Pdom = 0.646	−30.8 (−36.4 to −23.1)	Pdom = 0.500	NA	NA	NA	NA
	rs243849	CC	−8 (−10 to −6)	0.461	−31.8 (−39.8 to −24.7)	0.944	−6.5 (−7 to −4)	Padd = 0.535	−26.5 (−31.8 to −19)	Padd = 0.409
		CT + TT	−9.5 (−11 to −6)		−33.4 (−42 to −21.2)		−6 (−8 to −4)	Pdom = 0.797	−26.9 (−31.8 to −18.2)	Pdom = 0.576
	rs7201	AA	−8 (−9.8 to −6.3)	Padd = 0.896	−32.2 (−38.6 to −28.7)	Padd = 1.000	−6.5 (−7.3 to −4)	Padd = 0.878	−26.8 (−30.4 to −17.8)	Padd = 0.757
		AC	−8 (−11.5 to −6)		−34.8 (−41.7 to −18.7)		−7 (−8 to −4)		−26.9 (−33.3 to −19)	
		CC	−8 (−10 to −6)		−30.8 (−42.9 to −23.8)		−5.5 (−7.5 to −4)		−23 (−32.2 to −16.7)	
		AC + CC	−8 (−11 to −6)	Pdom = 0.788	−31.8 (−41.8 to −23.3)	Pdom = 0.992	−6 (−8 to −4)	Pdom = 0.983	−26.9 (−33.3 to −18.2)	Pdom = 0.866
*MMP9*	rs17576	AA	−7.5 (−10.3 to −5)	Padd = 0.620	−29.4 (−41 to −21.2)	Padd = 0.635	−6.5 (−7 to −4)	Padd = 0.780	−27.5 (−31.8 to −16.1)	Padd = 0.819
		AG	−8 (−11 to −6)		−31.8 (−41.9 to −28.6)		−6 (−8 to −4)		−25 (−33.3 to −18.6)	
		GG	−8 (−10 to −7)		−36 (−36 to −31.8)		−6.5 (−8.5 to −2.3)		−29 (−35 to −9.9)	
		AG + GG	−8 (−11 to −6.8)	Pdom = 0.346	−33.3 (−39.8 to −29)	Pdom = 0.349	−6 (−8 to −4)	Pdom = 0.488	−26.1 (−33.3 to −18.6)	Pdom = 0.530
	rs2250889	CC	−8 (−11 to −6)	0.379	−31.8 (−39.3 to −25)	0.587	NA	NA	NA	NA
		CG + GG	−6 (−10.5 to −3.5)		−27.3 (−45.8 to −12.7)		NA	NA	NA	NA
	rs17577	GG	−8 (−10 to −6)	0.558	−30.9 (−40.4 to −24.1)	0.718	−6 (−7 to −4)	0.498	−26 (−31.8 to −18.5)	0.516
		GA + AA	−9.5 (−11.8 to −4)		−36 (−40.7 to −18.7)		−7 (−8.5 to −4)		−26.9 (−34.1 to −17.8)	
	rs20544	CC	−8.5 (−11.3 to −7.8)	Padd = 0.385	−36 (−39.1 to −31.6)	Padd = 0.452	−6 (−8 to −0.5)	Padd = 0.475	−26.1 (−33.9 to −2.3)	Padd = 0.491
		CT	−8 (−10.5 to −6)		−30.8 (−41.5 to −21.4)		−6.5 (−8 to −4)		−27.7 (−33.3 to −19)	
		TT	−8 (−10 to −5.5)		−30.8 (−38.6 to −22.6)		−6.5 (−7 to −4)		−26 (−31.5 to −14.9)	
		CT + TT	−8 (−10.3 to −6)	Pdom = 0.168	−30.8 (−40.9 to −22.9)	Pdom = 0.210	−6.5 (−8 to −4)	Pdom = 0.488	−26.9 (−33.3 to −18.8)	Pdom = 0.590
*MMP3*	rs3025058	--	−6.5 (−12.8 to −4.5)	Padd = 0.777	−27.4 (−42.6 to −18.6)	Padd = 0.470	−6.5 (−7 to −5.3)	Padd = 0.889	−28.9 (−30.8 to −22.2)	Padd = 0.917
		-T	−8 (−11 to −6)		−36.0 (−41.7 to −26.1)		−7 (−8 to −4)		−26.9 (−33.3 to −17.0)	
		TT	−8 (−9 to −6.5)		−31.0 (−36 to −27.6)		−5 (−8 to −4)		−21.3 (−33.3 to −18.4)	
		-T + TT	−8 (−10 to −6)	Pdom = 0.562	−32.6 (−40.4 to −27.5)	Pdom = 0.501	−6 (−8 to −4)	Pdom = 0.898	−26.1 (−33.3 to −17.8)	Pdom = 0.943

Add—additive model; dom—dominant model; NA—data not available; these SNPs were not in agreement with the HWE.

## Data Availability

All the supporting data are reported in the Supplementary Files. All relevant raw data are available from the corresponding authors upon reasonable request.

## Supplementary Materials

The following supporting information can be downloaded at: https://www.mdpi.com/article/10.3390/ijms252413464/s1.
