# Supplementary material for: Association of Matrix Metalloproteinases Polymorphisms with Glaucoma Risk, Glaucoma Phenotype, and Response to Treatment with Selective Laser Trabeculoplasty or Latanoprost
Source: Int J Mol Sci. 2024 Dec 16;25(24):13464. doi: 10.3390/ijms252413464 (PMC11677773; doi:10.3390/ijms252413464)
Supplement: Supplementary file 1 [file ijms-25-13464-s001.zip › ijms-3311215-supplementary.pdf]

Table S1. Predicted SNP function, variant allele frequencies and assessment of Hardy-Weinberg equilibrium for selected MMP polymorphisms

Gene	SNP	Nucleotide / amino acid change	Location	Predicted SNP function*	VAF (healthy controls)	pHWE (healthy controls)	VAF (patients treated with latanoprost)	pHWE (patients treated with latanoprost)	VAF (patients treated with SLT)	pHWE (patients treated with SLT)
MMP14	rs1042703	p.Pro8Ser	Coding region	nsSNP	0.208	**0.037**	0.173	0.668	0.157	0.184
	rs1042704	p.Asp273Asn	Coding region	nsSNP, may influence splicing	0.201	0.903	0.298	0.801	0.186	0.256
	rs743257	c.*83C>T	3'-UTR	may influence miRNA binding, may alter chromatin states and regulatory motifs	0.509	0.359	0.462	0.606	0.461	0.509
MMP2	rs243865	c.-1306C>T	5'-UTR	may influence binding of transcription factors, may alter chromatin states	0.223	0.231	0.212	0.576	0.225	**0.038**
	rs243849	p.Asp333=	Coding region	may influence splicing	0.146	0.227	0.106	0.540	0.216	0.603
	rs7201	c.*260A>C	3'-UTR	differential miRNA binding, may alter regulatory motifs and tissue-specific gene expression	0.400	0.273	0.490	0.166	0.461	0.641
MMP9	rs17576	p.Gln279Arg	Coding region	nsSNP, may change protein function or structure, may influence splicing	0.357	0.322	0.356	0.800	0.324	0.392
	rs2250889	p.Arg574Leu	Coding region	nsSNP, may influence splicing	0.044	0.086	0.048	0.716	0.029	**<0.001**
	rs17577	p.Arg668Gln	Coding region	nsSNP, may influence splicing	0.155	0.087	0.087	0.284	0.167	0.153
	rs20544	c.*3C>T	3'-UTR	differential miRNA binding, may alter regulatory motifs and tissue-specific gene expression	0.558	0.424	0.567	0.881	0.647	0.407
MMP3	rs3025058	c.-1171 5A>6A	5'-UTR	may alter regulatory motifs	0.476	0.817	0.548	0.440	0.598	0.192

HWE – Hardy-Weinberg equilibrium, MMP – matrix metalloproteinase, ns – non-synonymous, SNP – single nucleotide polymorphism, SLT – selective laser trabeculoplasty, UTR – untranslated region, VAF – variant allele frequency

*evaluated using SNP function prediction, HaploReg and GTEx [51]

Table S2. Genotype frequencies of selected MMP polymorphisms

Gene	SNP	Genotype	Controls (N=339 subjects) N (%)	POAG and OHT patients (N=307) N (%)	Latanoprost-treated patients (N=52) N (%)	SLT-treated patients (N=51) N (%)
MMP14	rs1042703	TT	219 (64.6)	206 (67.1)	36 (69.2)	35 (68.6)
		TC	99 (29.2)	97 (31.6)	14 (26.9)	16 (31.4)
		CC	21 (6.2)	4 (1.3)	2 (3.8)	0 (0.0)
	rs1042704	GG	217 (64)	197 (64.2)	26 (50)	35 (68.6)
		GA	108 (31.9)	93 (30.3)	21 (40.4)	13 (25.5)
		AA	14 (4.1)	17 (5.5)	5 (9.6)	3 (5.9)
	rs743257	CC	86 (25.4)	67 (21.8)	16 (30.8)	16 (31.4)
		CT	161 (47.5)	158 (51.5)	24 (46.2)	23 (45.1)
		TT	92 (27.1)	82 (26.7)	12 (23.1)	12 (23.5)
MMP2	rs243865	CC	201 (59.3)	184 (59.9)	33 (63.5)	28 (54.9)
		CT	125 (36.9)	111 (36.2)	16 (30.8)	23 (45.1)
		TT	13 (3.8)	12 (3.9)	3 (5.8)	0 (0.0)
	rs243849	CC	250 (73.7)	218 (71)	42 (80.8)	32 (62.7)
		CT	79 (23.3)	80 (26.1)	9 (17.3)	16 (31.4)
		TT	10 (2.9)	9 (2.9)	1 (1.9)	3 (5.9)
	rs7201	AA	127 (37.5)	109 (35.5)	16 (30.8)	14 (27.5)
		AC	153 (45.1)	143 (46.6)	21 (40.4)	27 (52.9)
		CC	59 (17.4)	55 (17.9)	15 (28.8)	10 (19.6)
MMP9	rs17576	AA	136 (40.1)	121 (39.4)	22 (42.3)	22 (43.1)
		AG	164 (48.4)	149 (48.5)	23 (44.2)	25 (49.0)
		GG	39 (11.5)	37 (12.1)	7 (13.5)	4 (7.8)
	rs2250889	CC	311 (91.7)	278 (90.6)	47 (90.4)	49 (96.1)
		CG	26 (7.7)	28 (9.1)	5 (9.6)	1 (2.0)
		GG	2 (0.6)	1 (0.3)	0 (0.0)	1 (2.0)
	rs17577	GG	238 (70.2)	217 (70.7)	44 (84.6)	34 (66.7)
		GA	97 (28.6)	81 (26.4)	7 (13.5)	17 (33.3)
		AA	4 (1.2)	9 (2.9)	1 (1.9)	0 (0.0)
	rs20544	CC	70 (20.6)	56 (18.2)	10 (19.2)	5 (9.8)
		CT	160 (47.2)	153 (49.8)	25 (48.1)	26 (51.0)
		TT	109 (32.2)	98 (31.9)	17 (32.7)	20 (39.2)
MMP3	rs3025058	--	94 (27.7)	70 (22.8)	12 (23.1)	6 (11.8)
		-T	167 (49.3)	155 (50.5)	23 (44.2)	29 (56.9)
		TT	78 (23.0)	82 (26.7)	17 (30.7)	16 (31.4)

HWE - Hardy-Weinberg Equilibrium; VAF - Variant Allele Frequency.

Table S3. Association of MMP polymorphisms with glaucoma risk (POAG and OHT)

Gene	SNP	Genotype	Controls N (%)	Patients N (%)	OR (95% CI)	P	OR (95% CI) adj	Padj
MMP14	rs1042704	GG	217 (64)	197 (64.2)	Reference		Reference	
		GA	108 (31.9)	93 (30.3)	0.95 (0.68-1.33)	0.759	0.76 (0.44-1.32)	0.332
		AA	14 (4.1)	17 (5.5)	1.34 (0.64-2.78)	0.437	1.80 (0.55-5.89)	0.333
		GA+AA	122 (36)	110 (35.8)	0.99 (0.72-1.37)	0.967	0.85 (0.51-1.44)	0.555
	rs743257	CC	86 (25.4)	67 (21.8)	Reference		Reference	
		CT	161 (47.5)	158 (51.5)	1.26 (0.85-1.86)	0.243	0.88 (0.47-1.66)	0.697
		TT	92 (27.1)	82 (26.7)	1.14 (0.74-1.77)	0.546	1.04 (0.52-2.09)	0.914
		CT+TT	253 (74.6)	240 (78.2)	1.22 (0.85-1.75)	0.29	0.94 (0.52-1.70)	0.832
MMP2	rs243865	CC	201 (59.3)	184 (59.9)	Reference		Reference	
		CT	125 (36.9)	111 (36.2)	0.97 (0.70-1.34)	0.854	0.84 (0.49-1.42)	0.513
		TT	13 (3.8)	12 (3.9)	1.01 (0.45-2.27)	0.984	0.48 (0.13-1.77)	0.273
		CT+TT	138 (40.7)	123 (40.1)	0.97 (0.71-1.33)	0.868	0.79 (0.48-1.33)	0.378
	rs243849	CC	250 (73.7)	218 (71)	Reference		Reference	
		CT	79 (23.3)	80 (26.1)	1.16 (0.81-1.66)	0.416	1.11 (0.62-1.96)	0.728
		TT	10 (2.9)	9 (2.9)	1.03 (0.41-2.59)	0.946	1.86 (0.37-9.22)	0.449
		CT+TT	89 (26.3)	89 (29)	1.15 (0.81-1.62)	0.437	1.16 (0.66-2.01)	0.609
	rs7201	AA	127 (37.5)	109 (35.5)	Reference		Reference	
		AC	153 (45.1)	143 (46.6)	1.09 (0.77-1.53)	0.626	1.07 (0.61-1.87)	0.816
		CC	59 (17.4)	55 (17.9)	1.09 (0.69-1.70)	0.718	1.52 (0.75-3.10)	0.249
		AC+CC	212 (62.5)	198 (64.5)	1.09 (0.79-1.50)	0.606	1.18 (0.70-1.99)	0.524
MMP9	rs17576	AA	136 (40.1)	121 (39.4)	Reference		Reference	
		AG	164 (48.4)	149 (48.5)	1.02 (0.73-1.42)	0.901	0.77 (0.45-1.31)	0.328
		GG	39 (11.5)	37 (12.1)	1.07 (0.64-1.78)	0.806	0.60 (0.26-1.35)	0.216
		AG+GG	203 (59.9)	186 (60.6)	1.03 (0.75-1.41)	0.855	0.73 (0.44-1.21)	0.218
	rs2250889	CC	311 (91.7)	278 (90.6)	Reference		Reference	
		CG+GG	28 (8.3)	29 (9.4)	1.16 (0.67-2.00)	0.596	0.99 (0.40-2.48)	0.985
	rs17577	GG	238 (70.2)	217 (70.7)	Reference		Reference	
		GA	97 (28.6)	81 (26.4)	0.92 (0.65-1.30)	0.620	0.78 (0.44-1.37)	0.383
		AA	4 (1.2)	9 (2.9)	2.47 (0.75-8.13)	0.137	1.81 (0.30-11.02)	0.520
		GA+AA	101 (29.8)	90 (29.3)	0.98 (0.70-1.37)	0.894	0.83 (0.48-1.43)	0.492
	rs20544	CC	70 (20.6)	56 (18.2)	Referenca		Referenca	
		CT	160 (47.2)	153 (49.8)	1.20 (0.79-1.81)	0.400	1.18 (0.60-2.33)	0.636
		TT	109 (32.2)	98 (31.9)	1.12 (0.72-1.75)	0.607	1.45 (0.71-2.97)	0.308
		CT+TT	269 (79.4)	251 (81.8)	1.17 (0.79-1.72)	0.441	1.29 (0.68-2.43)	0.439
MMP3	rs3025058	--	94 (27.7)	70 (22.8)	Referenca		Referenca	
		-T	167 (49.3)	155 (50.5)	1.25 (0.85-1.82)	0.255	1.46 (0.78-2.75)	0.241
		TT	78 (23.0)	82 (26.7)	1.41 (0.91-2.19)	0.123	1.92 (0.94-3.93)	0.073
		-T+TT	245 (72.3)	237 (77.2)	1.30 (0.91-1.86)	0.155	1.60 (0.88-2.91)	0.121

Adj: Adjustment for age and sex
